# Kenyan adults with type 2 diabetes mellitus (T2DM) increase diabetic knowledge and self-efficacy and decrease hemoglobina1c levels post-educational program

**DOI:** 10.4314/ahs.v24i1.21

**Published:** 2024-03

**Authors:** Sabina Jeruto Bett, Jochebed Bosede Ade-Oshifogun

**Affiliations:** 1 School of Nursing, Andrews University, 8475 University Blvd, Berrien Springs, MI, 49104. USA; 2 School of Nursing, Andrews University, 8475 University Blvd, Berrien Springs, MI, 49104. USA; 3 School of Nursing, College of HEST, P.O.Box 3001, New Mexico State University, Las Cruces, NM 88003

**Keywords:** Education program, type 2 diabetes, Kenya

## Abstract

**Introduction:**

Literature supports the relationship between increased diabetic knowledge and improved health outcomes among individuals with Type II diabetes mellitus (T2DM). In Kenya, knowledge gaps within the at-risk population still exist about the symptoms, complications, and management strategies of T2DM, making it challenging to achieve the required personal and community health levels. The project's objective was to determine whether a structured educational intervention for patients in Eldoret, Kenya, would increase diabetic knowledge and self-efficacy and reduce HbA1c levels.

**Method:**

We utilized an experimental study with a convenience sample of 143 participants systematically grouped into control and experimental. The experimental group only received a structured educational intervention based on the health belief model. Pre- and post-intervention data for diabetic knowledge, self-efficacy, and HbA1c were analyzed using the independent T and ANOVA tests.

**Results:**

We observed significant between-group differences for diabetic knowledge (t (116) = 7.22, p<0.001), self-efficacy t (96)=5.323, p<0.001; and HbA1c level t (121) =−2.87, p =.003. We also observed significant within-group differences for diabetic knowledge, t (12.6), p<0.001); self-efficacy t (5.32), p<.001); and HbA1c, t (4.4), p<0.001, in the experimental group only.

**Conclusions:**

This study reveals the effect of a structured education intervention in increasing diabetic knowledge and self-efficacy while reducing HbA1c levels in T2DM patients in Eldoret, Kenya.

## Introduction

Type II diabetes mellitus (T2DM) is a chronic and non-communicable disease, with about 1.4 million people 18 years or older in the USA having this diagnosis in 2019^1^. The probability of Kenyans between 30 and 70 years dying from diabetes is 21 percent^2^. The International Diabetes Federation (IDF) in 2021 estimated that 24 million 20 – 79 years-old adults living in the IDF African region, including Kenya, have diabetes, with 54% undiagnosed^3^. The prevalence of diabetes in Kenya standardized by age was 2.4%, with 44% being aware of their condition and only 7% controlling blood sugar^4^. Although health outcomes in Kenya have improved since 2006 due to a decrease in the burden of communicable diseases, non-communicable diseases such as diabetes have increased^5^. The need to educate Kenyan adults about diabetic risk factors and diabetic-related complications increases with the increasing prevalence of diabetes among this population.

Few research studies addressed the need for diabetic education in Kenya. A significant factor contributing to the increased morbidity from T2DM in Kenya is the diabetic knowledge gap. The risk reduction knowledge gap was identified in prediabetic patients in Kenya^6^. Patients usually seek diabetic care late after developing severe and irreversible complications^6^. Several studies document the positive impact of diabetic education on positive health outcomes^7^,^8^. The Kenyan government has sought to eliminate the negative impact by developing the national diabetes strategy and the Kenya National Diabetes Educators Manual. However, these initiatives are yet to be evaluated 9. The few studies addressing diabetic education in Kenya identified the following factors a) a lack of educational efforts 9; b) the concept of managing T2DM by using HbA1c is not prevalent 9; c) perceived high cost of testing HbA1c levels in Africa 10; and d) significant gap in policy at the community levels. Other studies identified low diabetic dietary and comorbidity knowledge 11,12 as knowledge gap areas. Other factors identified were the impact of cultural practices 13,14; and self-care practices 15. These knowledge gaps are critical areas of T2DM management, hindering the fight against T2DM in Kenya.

This project utilized the Health Belief Model (HBM) for its theoretical approach. According to the model, health behavior can be explained by the influence of modifying factors on individual perceptions to produce a given action or health outcome 16. The generation and application of new knowledge for a given health outcome are enhanced through health education and behavior. The HBM is constructed around six primary constructs – perceived susceptibility, perceived severity, perceived benefits, perceived barriers, self-efficacy, and cues to action 16. In the HBM, an individual's motivation for a health behavior is categorized as individual perceptions, modifying factors, and the likelihood of action 17. Following the assumptions and constructs of the model, the project assumed that by introducing educational intervention as a modifying factor, individual perceptions about diabetes would change. To make it effective, we structured the educational intervention within the cultural context and food preferences. The predicted outcome includes improved self-efficacy, increased diabetic knowledge, and reduced HbA1c levels.

The American Association of Clinical Endocrinologists (AACE) position statement supports the use of culturally appropriate education that focuses on the critical knowledge areas to alleviate the challenges of managing diabetes and increasing self-efficacy 18. The culturally relevant educational models allow the individuals to identify practices that may influence their HbA1c levels and self-care practices 19. Different education curricula and structures affect the effectiveness of interventions differently. Patient-to-patient education was reported to result in higher glycemic control 20. Other authors used a formal educational structure with a specified timeframe for the intervention 21; and a nurse-led design 19. Additionally, group educational models had significant advantages that would benefit the Kenyan population because of the cost-effectiveness and enhanced collaboration between stakeholders 22.

In summary, knowledge is a significant modifying variable and can enhance self-care and perception. The structure of the educational intervention similarly is a determining factor for the outcome. Knowledgeable individuals will likely make health-seeking decisions, including dietary changes, activity and exercise, and adherence to treatments and intervention plans. Effective health-seeking decisions are achievable when a patient has high levels of self-efficacy. This project aimed to increase diabetic knowledge and self-efficacy through culturally appropriate educational intervention to decrease HbA1c levels in Kenyan people with T2DM.

## Design, materials & method

### Design and protocol

**Figure 1:**
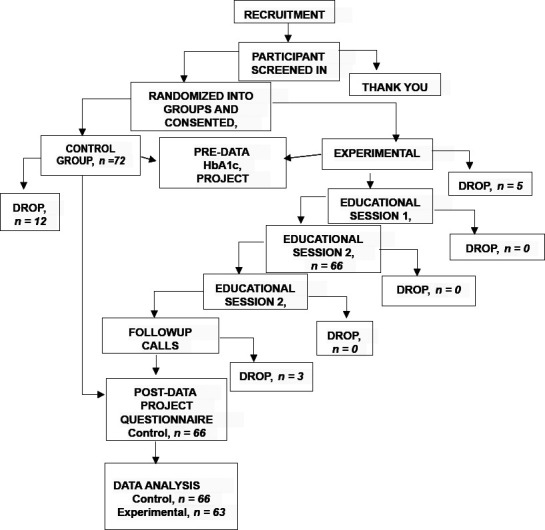
Project design and protocol

A quasi-experimental design was utilized for this project. The participants were divided into a control and an experimental group by systematic assignment. The participants in the experimental group were enrolled in a three-month diabetic educational program provided by one of the authors. The following figure 1 depicts the project design and protocol.

### Setting

Eldoret is a cosmopolitan Kenyan city located in Western Kenya. The city has a diverse population but a significantly larger Kalenjin population. The community hospital (Reale Hospital), used for participant recruitment, is a well-equipped, 500 beds private hospital located within Eldoret City and serves patients from urban, suburban, and rural locations around the city. The hospital provides inpatient and outpatient services. The hospital serves an average of 750 patients a day, out of which about 100 have a diagnosis of T2DM. Because of time constraints and costs, the hospital provides limited diabetic education to this population.1

### Sample

The sample size was estimated with the G*Power Software (Version 3.1). One hundred and forty-three subjects were recruited by convenience sampling. The participants were screened for inclusion with the following criteria: a history of T2DM; HbA1c ≥ 6.5% for one year or more via chart review; African heritage; ability to read, write, understand, and speak in English or Swahili; and age between 25–65 years. The educational program was provided in English and Swahili. The age range was selected to eliminate bias from third parties' influence on project outcomes, especially with elderly patients' dependence on caretakers. People with pre-existing conditions that may complicate T2DM, like cancer, mental illness, and any current illness, were excluded from participation. The participants were systematically assigned to experimental (n=71) and control groups (n=72). Participants completed a written consent after receiving a full explanation of the protocol. Incentives given to increase participation includes “The Plate” guide used for portion control, the meal planning guidebook, transportation expense reimbursement (10 dollars), and subsidized HbA1c tests. The HbA1c testing cost was 80 percent funded by Reale Hospital to support the project.

### Ethics

We obtained ethics approval from the Andrews University IRB, the University of East Africa Baraton REC, the National Commission for Science Technology, Innovation (NACOSTI), Reale Hospital, and the Uasin Gishu County Government.

### Project tools and variables

We measured three main variables - diabetic knowledge, self-efficacy, and HbA1c levels. We utilized the University of Michigan Diabetic Knowledge Test (DKT) to measure Diabetic Knowledge (DK) 23. The test has 25 questions about an individual's knowledge of diabetic disease and management, focusing on the diabetic diet, diabetic testing, recognizing complications of DM, and self-management. With permission from the authors, two questions were added to the 23 items on the Michigan DKT, and five questions were modified to fit the cultural context of the project settings. The modification and addition did not change the concepts measured by the tool. The tool was scored with participants obtaining one point for each correct answer, with 25 possible points. For this project, the cut-off point for adequate diabetic knowledge was a score of 20 out of 25. The tool has consistent reliability data with a ≥ 0.70 24.

Self-efficacy was defined in this project as a participant's confidence in their ability to manage T2DM. We measured self-efficacy levels with the Stanford University Diabetes Questionnaire (SUDQ) 25. The questionnaire has eight items, scored on a scale of 1 (lowest confidence) to 10 (highest confidence). The score determines the individual's belief in self-managing T2DM. The average score of all eight items is considered the overall self-efficacy. In this project, an average score of 7 was regarded as adequate self-efficacy. The internal consistency of the SUDQ was 0.89, and the intraclass correlation coefficient was 0.90 25.

Reale Hospital laboratory technicians assisted with measuring the participants' HbA1c levels using the Afinion™ HbA1c1 assay (Abbott). Demographic data include gender, age, education, marital status, tribe, occupation, income, and household size.

### Educational program

The educational program was structured to bring awareness about a balanced diet as beneficial in managing an individual's blood glucose levels. In this project, we defined a balanced diet as consuming food items from the four main food groups: carbohydrates, protein, fats, and vegetables. We used the plate model to instruct participants because it was easy to use and helpful in controlling portion sizes. The food groups were related to culturally appropriate foods in Kenya. We used participants' individual goals to focus on and visualize the benefits of dietary compliance. The objectives of the educational intervention were to increase diabetic knowledge and self-efficacy and reduce HbA1c.

We structured the intervention into three modules taught over three weekly sessions for the experimental group. Participants could join any three consecutive sessions within the three-month intervention period. The first two-hour sessions focused on general diabetes knowledge education, including symptoms, complications, medication, and the significance of HbA1c testing. The participants discussed their self-care goals and identified barriers to achieving the goals.

The participants learned about T2DM management in the second session, including nutrient calculations from food labels and aligning meals with the “MyPlate” method. We used the ADA guidelines to instruct participants about portion sizes by requesting they create an imaginary line in the 9-inch plate 26. They were asked to fill the first half of the plate with culturally appropriate non-starchy vegetables like sukuma (collard greens), sucha (black midnight), and isaka (spider flower) terrier (amarantha). The other half of the plate was divided into two quarters - one-quarter of carbohydrates and a quarter of protein. Examples of protein-rich foods in Kenya include nyama choma (roasted meat), ndengu (lentils), maharakwe (beans), milk, and eggs. Carbohydrate foods include ugali (corn meal), rice, mokimo (a mixture of mashed potatoes, beans, greens, and corn), pumpkins, yam, and cassava. Participants can vary their meal plans to include servings of fruits and milk to substitute for carbohydrates and protein. Participants can have liberal amounts of unsweetened drinks such as tea, coffee, or water. The participants revised their goals after discussing and learning the benefits of dietary education and the barriers to adherence. All participants included goals to mitigate the barriers to compliance.

We focused the third session on coping with T2DM and the role of family and community members in managing diabetes. Participants also discussed the perceived benefits of the education, barriers, and cues to action.

### Data analysis

Descriptive statistics were used for the demographic data. Between and within-group analysis was computed using Statistical Package for the Social Sciences (SPSS, v.25). Paired sample t-test was used for within-group pre and post-test data analysis. Data comparisons between experimental and control groups were computed with an independent T-test after ensuring homogeneity of variance by Levene's Test. The normality of the dataset was assessed using the Shapiro-Wilk test. We compared data from diabetic knowledge, self-efficacy, and HbA1c pre- and post-intervention. The level of significance was set at p < 0.05.

## Results

One hundred and twenty-three (123) participants completed the study. There were 63 participants in the experimental and 60 in the control group. The demographic data is depicted in Table 1 below. There were slightly more female participants (57%) in the control group compared to the experimental group (48%); however, the difference was not significant (p =.315). The largest tribe in the study was the Kalenjin, representing 61% of the participants.

**Table 1:** Demographic characteristics

Variable	Control (n=60) f (%)	Experimental (n=63) f (%)	Total (n=123) f (%)
**Gender**			
Male	26 (43.3)	33 (52.4)	59 (48.0)
Female	34 (56.7)	30 (47.6)	64 (52.0)
**Age group**			
25 – 34	1 (1.7)	2 (3.2)	3 (2.4)
35 – 44	7 (11.7)	7 (11.2)	14 (11.4)
45 – 54	18 (30.0)	23 (36.5)	41 (33.3)
55 - 65	34 (56.7)	31 (49.2)	65 (52.8)
**Occupation**			
Teacher	20 (33.3)	17 (27.0)	37 (30.1)
Farmer	22 (36.7)	17 (27.0)	39 (31.7
Doctor	0 (0)	1 (.6)	1 (0.8)
Shopkeeper	2 (3.3)	1 (1.6)	3 (2.4)
Other	16 (26.7)	24 (38.1)	40 (32.5)
**Education**			
Primary School	12 (20)	16 (25.4)	28 (22.8)
Secondary school	11 (18.3)	14 (22.2)	25 (20.3)
College/university	30 (50)	29 (46.0)	59 (48)
Graduate	7 (11.7)	1 (1.6)	8 (6.5)
**Marital status**			
Married	47 (78.3)	52 (82.5)	99 (80.5
Separated	6 (10.0)	5 (7.9)	11 (8.9)
Widowed	1 (1.7)	2 (3.2)	3 (2.4)
Single Gender	5 (8.3)	4 (6.3)	9 (7.3)
**Income**			
< KSH 25,000	25 (41.7)	31 (49.2)	56 (45.5)
KSH 25,000 - 50,000	21 (35)	15 (23.8)	36 (29.3)
	8 (13.3)	7 (11.1)	15 (12.2)
> KSH 50,000	2 (3.3)	5 (7.9)	7 (5.7)
Other	4 (6.7)	4 (6.3)	8 (6.5)
I do not know			
**Tribe**			
Kalenjin	42 (70)	33 (52.4)	75 (61.0)
Luo	2 (3.33)	2 (3.2)	4 (3.3)
Luyha	7 (11.7)	11 (17.5)	18 (14.6)
Kikuyu	6 (10)	13 (20.6)	19 (15.4)
Other	3(5)	4(6.3)	7(5)

The Shapiro-Wilk test yielded a test statistic (W) of 0.987 and a corresponding p-value of 0.338. The p-value was greater than our predetermined significance level of 0.05 suggesting that the data may be normally distributed. Analysis of pre-intervention data for between-group differences showed no significant results for diabetic knowledge (t (121) =-1.180, p=.120); self-efficacy (t (121) =0.962, p=.169); and HbA1c levels (t (121) =-0.426, p=.336). However, as depicted in Table 2, the between-group differences for post-intervention scores were significant for diabetic knowledge (t (116) =7.218, p<.001); self-efficacy (t (96) =5.323, p<.001); and HbA1c (t (121) =-2.87, p=.003). Diabetic knowledge was significantly improved between pre- and post-data in both groups (control, p=.037; experimental, p<.001).

**Table 2:** Between group data for experimental and control groups Control Experimental

Variable	(n= 60)		(n= 63)		Test Statistics	*p* *

	M	SD	M	SD		
Pre-HbA1c	9.11	2.19	9.30	2.64	*t* (121) =-0.426	.336
Post-HbA1c	9.13	2.22	8.07	1.89	*t* (121) =-2.87	.003**
Pre-Self efficacy	6.08	1.92	6.37	1.43	*t* (121) =0.962	.169
Post-Self efficacy	6.37	1.82	7.94	1.31	*t* (96) =5.323	<.001**
Pre-Diabetes	15.08	3.524	14.33	3.524	*t* (121) =-1.180	0.120
Knowledge Test						
Post-Diabetes	15.80	3.509	20.44	3.468	*t* (116) =7.218	<0.001**
Knowledge Test						

* 1 tailed (α = 0.025)

** *p* values are significant

The mean HbA1c levels in the control group slightly increased from 9.11±2.19 to 9.13± 2.22, but the increase was not significant. However, although there was a significant difference in the experimental group's pre- and post-HbA1c levels (p<.001), the control group's pre- and post-data was not significantly different (p=.467). Participants in the experimental group significantly increased their self-efficacy scores by a mean of 1.57 (p<.001). All post-intervention data were substantially different from the pre-intervention data in the experimental group. In contrast, the only significant difference in scores from the control group was diabetic knowledge (see Table 3).

**Table 3:** Within group data for experimental and control groups

Variable					Pre-test	Post
		
	M	SD	M	SD	*t*-test	*p*
Diabetes knowledge score in experimental group	14.33	3.524	20.44	3.47	*t* (62) =12.604,	<.001**
Diabetes knowledge score in control group	15.18	3.502	15.80	3.51	*t* (62) =1.824,	.037**
Self-efficacy score in experimental group	6.37	1.43	7.94	1.31	*t* (62) = -7.29	<.001**
Self-efficacy score in control group	6.10	1.87	6.37	1.82	*t* (54) = -.984	.165
HbA1c in the experimental group	9.30	2.64	8.07	1.89	*t* (62) =4.396,	<0.001**
HbA1c in the control group	9.11	2.19	9.13	2.22	*t* (59) =-0.083	.467

** *p* values are significant

## Discussion

The educational program structured to influence the individual participants' perception as per the HBM showed a significant effect on diabetic knowledge, self-efficacy, and HbA1c levels. Diabetic self-management interventions have generally been shown to improve physiological outcomes in Africans 27. Self-awareness of treatment targets and self-blood glucose monitoring were among the important factors associated with successful diabetic control in a study conducted in a university clinic 28. Patient education and training have also improved chronic disease control, such as T2DM 28; and hypertension 29. Our results support the need to improve self-management through structured education and increased self-efficacy for Kenyan adults with T2DM. Although our study did not address comorbidities; however, we realize the multiple challenges faced with managing T2DM with the comorbidities that will require careful adjustment to standard diabetic education and disease management training. The comorbidities include heart diseases 30 and renal impairment 31. The level of formal education in Kenya continues to improve slowly compared to other developed countries. The slow improvement is also evident in the health education of individuals and communities. The older Kenyan population (45 years and older), with a higher risk of T2DM, was reported to have little or limited formal education 4. However, most participants in this study (62%) had a college or graduate degree. The inclusion criteria of reading and writing may explain our research's increased number of well-educated participants. The higher percentage of married participants in our study (81%) was close to 67% reported in the national survey 4. The improved self-efficacy and lower HBA1c may be attributed to a supportive spouse at home helping with the dietary changes.

Kenya is a multi-tribal country, and the Kalenjins have been identified as the 6th most prominent tribe in the country 32. Many Kalenjins live in and near Eldoret, which accounts for many participants from this tribe. However, the diet is similar between tribes. Substituting Kenyan food with the ‘my plate’ method for nutritional education was an eye-opener for the participants as they believed removing table sugar from their diet was all they needed to manage T2DM. In this project, the participant's perceptions of dietary and lifestyle changes were influenced by diabetic education, as evidenced in the post-education increased self-efficacy. Improving patient knowledge with patient education has beneficial effects on diabetic control and reducing diabetic complications 31. Although we did not measure self-management, their increased self-efficacy contributed to subsequent disease self-management resulting in decreased HbA1c levels observed in the experimental group. The lack of change in the control group's self-efficacy and HbA1c levels further validates the effect of the educational program. Our pre-intervention HbA1c was significantly different from the post-intervention HbA1c, contrasting with the findings from a non-blinded randomized clinical trial in Nairobi, Kenya 1. Diabetic knowledge has been a critical factor in glycemic control among T2DM patients. The lack of significant difference in the control group's pre- and post-diabetic knowledge test scores attests to the general community's knowledge deficit about T2DM.

The limitations of the study include seasonal timing. The study took place during the festive season in Kenya (November-December). We could achieve lower HbA1c levels if the study were completed during a non-festive season with less urge to eat more. The participants' education could account for greater comprehension and motivation for dietary compliance. Using a single site for this study is a limitation, as the findings may differ from other sites with a diverse patient population. Although we recruited and educated participants over three months, the relatively small number of participants also limits the generalizability of this study. We suggest a future study with a larger sample size across multiple sites to decrease these limitations. The single site may also account for the attrition observed in the study. Because of the location and size of the hospital serving both urban and rural communities, the long commute to the hospital may be challenging for patients that live far resulting in their inability to return for post-intervention measures. Another limitation was the modified Michigan DKT questionnaire was not pilot tested for reliability, although the authors approved the changes to the questionnaire as appropriate. This face validity can be strengthened through pilot testing. Although paired t-tests for within-group pre-and post-test data analysis are a potential source of type 1 error, our current analysis aligns with our study's specific objectives and research questions, and we have confidence in the validity of our findings based on this approach.

## Conclusion

The findings from this study suggest that a structured diabetic educational program improves HbA1c levels, diabetic knowledge, and self-efficacy in Kenyan people with T2DM. We recommend public awareness and increase structured diabetic education in Kenyan hospitals and community settings to improve health outcomes for people with T2DM.

## References

[1] CDC (2022). A snapshot: Diabetes in the United States [Internet].

[2] WHO (2022). WHO region and global statistics [Internet].

[3] IDF (2022). IDF Africa Region [Internet].

[4] Mohamed SF, Mwangi M, Mutua MK, Kibachio J, Hussein A, Ndegwa Z (2018). Prevalence and factors associated with pre-diabetes and diabetes mellitus in Kenya: Results from a national survey. BMC Public Health.

[5] Achoki T, Miller-Petrie MK, Glenn SD, Kalra N, Lesego A, Gathecha GK (2019). Health disparities across the counties of Kenya and implications for policymakers, 1990–2016: a systematic analysis for the Global Burden of Disease Study 2016. Lancet Glob Health.

[6] Munyasia LN, George A, Amimo F, Wafula SW (2021). Diabetes Awareness and Risk Reduction Behaviors among Prediabetic Patients in Busia County, Western Kenya.

[7] Abd-El mohsen SA, Mohamed Shehata AA (2020). Evaluating the effect of health education program on outcomes of type I diabetic patients: A randomized controlled study. Saudi J Btol Sci.

[8] Hamideh Lari, Rahim Tahmasebi, Azita Noroozi (2018). Effect of electronic education based on health promotion model on physical activity in diabetic patients.

[9] Jones T LE (2013). Diabetes Mellitus: the increasing burden of disease in Kenya. South Sudan Med J.

[10] George J (2011). Should haemoglobin A1c be used for the diagnosis of diabetes mellitus in South Africa?. J Endocrinol Metab Diabetes South Afr.

[11] Marwa I, Mtshali G (2022). Challenges in the Management of Chronic Comorbid Conditions (Diabetes and Hypertension) in Kenya.

[12] Wahome EM, Makau WK, Kiboi WK (2018). Predictors of dietary practices and nutritional status among diabetic type II patients in Kiambu County, Kenya. Int J Community Med Public Health.

[13] Ngari DM, Mbisi AM, Njogu TW (2020). Social, Cultural and Economic Factors Affecting the Practice of Secondary Prevention among Patients with Type 2 Diabetes Mellitus at Consolata Nkubu and Meru Level Five Hospital in Meru County. Open J Clin Diagn.

[14] Lloyd CE, Musyimi C, Mutiso V, Ndetei D (2022). Individual and community experiences and the use of language in understanding diabetes and depression in rural Kenya. Glob Public Health.

[15] Kyallo EGWF, Kiage B (2020). Knowledge and self-care practices among diabetic patients: A case study of Thika level 5 hospital, Kenya. J Health Med Nurs.

[16] Jiang L, Liu S, Li H, Xie L, Jiang Y (2021). The role of health beliefs in affecting patients' chronic diabetic complication screening: a path analysis based on the health belief model. J Clin Nurs.

[17] Mckellar K, Sillence E (2020). Current research on sexual health and teenagers: Health belief model. Teenagers, Sexual Health Information and the Digital Age.

[18] Mechanick JI, Adams S, Davidson JA, Fergus I V, Galindo RJ, McKinney KH (2019). Transcultural diabetes care in the United States – A position statement by the American Association of Clinical Endocrinologists. Endocr Pract.

[19] Whitehead LC, Crowe MT, Carter JD, Maskill VR, Carlyle D, Bugge C (2017). A nurse-led education and cognitive behaviour therapy- based intervention among adults with uncontrolled type 2 diabetes: A randomized controlled trial. J Eval Clin Pract.

[20] Xiang Y, Luo P, Cai X, Tang Y, Wu Z (2017). Results of a pilot study of patient-to-patient education strategy on self-management among glycemic uncontrolled patients with diabetes. Patient Prefer Adherence.

[21] Lamptey Roberta, Robben MP, Coleman MA, Boateng D, Grobbee DE, Davies MJ (2022). Structured diabetes self-management education and glycaemic control in low-and. pdf. Diabet Med.

[22] Steinsbekk A, Rygg L, Lisulo M, By Rise M, Fretheim A (2012). Group based diabetes self-management education compared to routine treatment, waiting list control or no intervention for people with type 2 diabetes mellitus. BMC Health Serv Res.

[23] Collins GS, Mughal S, Barnett AH, Fitzgerald J, Lloyd CE (2010). Modification and validation of the Revised Diabetes Knowledge Scale. Diabet Med.

[24] Fitzgerald JT, Funnell MM, Hess GE, Barr PA, Anderson RM, Hiss RG (1998). The reliability and validity of a brief diabetes knowledge test. Diabetes Care.

[25] Lorig K, Ritter PL, Villa FJ, Armas J (2009). Community-Based Peer-Led Diabetes Self-management. Diabetes Educ.

[26] Chang SJ, Song M, Im EO (2014). Psychometric evaluation of the Korean version of the Diabetes Self-efficacy Scale among South Korean older adults with type 2 diabetes. J Clin Nurs.

[27] American Diabetes Association (ADA) (2018). Classification and diagnosis of diabetes: Standards of medical care in Diabetesd2018. Diabetes Care.

[28] Kocak MZ, Aktas G, Erkus E, Duman T, Atak BM, Savli H (2018). Analysis of the Type 2 Diabetic Patients Followed in a University Clinic. Konuralp Medical Journal.

[29] Atik F, Aktas G, Kocak MZ, Erkus E, Savli H (2018). Analysis of the factors related to the blood pressure control in hypertension. J Coll Physicians Surg Pak.

[30] Aktas G, Atak Tel BM, Tel R, Balci B (2023). Treatment of type 2 diabetes patients with heart conditions. Expert Rev Endocrinol Metab.

[31] Bilgin S, Kurtkulagi O, Duman TT (2022). Sodium glucose co-transporter-2 inhibitor, Empagliflozin, is associated with significant reduction in weight, body mass index, fasting glucose, and A1c levels in Type 2 diabetic patients with established coronary heart disease: the SUPER GATE study. Ir J Med Sci.

[32] Diriba DC, Leung DYP, Lorna KP (2020). Suen. The effects of diabetes self-management interventions on physiological outcomes in people living with diabetes in Africa: A systematic review and meta-analysis. Diabet Med.

